# On the Pseudo Phase Diagram of Single Semi-Flexible Polymer Chains: A Flat-Histogram Monte Carlo Study

**DOI:** 10.3390/polym9020038

**Published:** 2017-01-25

**Authors:** Benno Werlich, Mark P. Taylor, Timur Shakirov, Wolfgang Paul

**Affiliations:** 1 Institut für Physik, Martin-Luther-Universität, 06099 Halle, Germany; Benno.Werlich@physik.uni-halle.de (B.W.); Timur.Shakirov@physik.uni-halle.de (T.S.); 2 Department of Physics, Hiram College, Hiram, OH 44234, USA; taylormp@hiram.edu

**Keywords:** semi-flexible polymers, Stochastic Approximation Monte Carlo, pseudo phase diagram, ground-state morphology

## Abstract

Local stiffness of polymer chains is instrumental in all structure formation processes of polymers, from crystallization of synthetic polymers to protein folding and DNA compactification. We present Stochastic Approximation Monte Carlo simulations—a type of flat-histogram Monte Carlo method—determining the density of states of a model class of single semi-flexible polymer chains, and, from this, their complete thermodynamic behavior. The chains possess a rich pseudo phase diagram as a function of stiffness and temperature, displaying non-trivial ground-state morphologies. This pseudo phase diagram also depends on chain length. Differences to existing pseudo phase diagrams of semi-flexible chains in the literature emphasize the fact that the mechanism of stiffness creation matters.

## 1. Introduction

Local stiffness or semi-flexibility of polymer chains is probably by far the most important design concept for the creation of ordered polymeric structures. This is obviously true for (semi-)crystalline synthetic polymers which order into lamellar structures [1], displaying not only positional order of the monomers but also orientational order of the chains. Similarly, nature makes use of stiffness of bio-polymers in processes such as the encapsulation of DNA [2] and the formation of secondary structure (α-helices, β-sheets) in proteins [3], as well as in the formation of pathogenic amyloid [4].

Theoretical and computational modeling of semi-flexible chains started very early on, e.g., by Flory’s approach to model the isotropic-nematic transition in semi-flexible polymers using a simple lattice model [5]. The most important theoretical model is certainly the worm-like chain [6] with its prediction of an exponential decay of bond-orientation correlation along the chain on the scale of its persistence length. The property of this model, that the Kuhn-length of a chain is twice its persistence length, has become such common folklore that it is often taken as a general relation, although it is only valid within this model of a persistent type of semi-flexibility. The fact that not all stiffnesses are created equal has been stressed and clearly worked out in the theoretical analysis of the isotropic-nematic transition of melts of semi-flexible chains [7]. In addition, in recent years, it has been established by simulations that predictions of the worm-like chain model are actually never realized in nature due to the neglect of excluded volume interactions in the model. Orientational correlations within a chain asymptotically always decay algebraically [8,9,10,11,12] and not exponentially.

In a computer simulation of semi-flexible chains, the persistent mechanism of flexibility is most easily realized by a bond angle potential that favors zero degrees in its ground state. Such a modeling approach has been used to explain the occurrence of toroidal structures in the collapsed state of stiff DNA chains [13,14]. The phase diagram of this type of model as a function of stiffness and non-bonded attraction was first studied by Grassberger [15] for the simple cubic self-avoiding walk, where it was established that the collapse transition of a polymer chain becomes a first order transition beyond a certain stiffness. The pseudo phase diagram at finite chain length was studied for the bond-fluctuation model (which has 87 different bond angles compared to 2 in the simple cubic lattice model) using extended ensemble Monte Carlo simulations [16,17,18] and was shown to exhibit regions of stability for lamellar structures, toroidal structures and tennis racket like structures (a combination of the two former ones). While these studies were performed for chains of lengths N=40−256, a Wang–Landau Monte Carlo [19,20] study of such semi-flexible bead-spring chains in the continuum, performed for chains of length N=13−55 [21] later confirmed the occurrence of these morphologies. Recently a similar model was studied by multi-canonical simulations [22,23], and it was shown that ground states at different stiffness can also be characterized by their knottedness (here, knots are defined by joining the two ends of the chain by an additional long bond).

A different mechanism of introducing stiffness was suggested for the modeling of proteins. In one variant, chains described as thick tubes were suggested [24,25], which, upon collapse, are able to form helical ground states. In a different variant [26,27], the chains were built out of overlapping hard spheres, which, upon collapse, exhibit helical ground states for short enough chains. This model is promising for a systematic study of the effect of such steric stiffness on the single chain phase diagram [28], and we will report in the following on such a study.

## 2. Materials and Methods

**Figure 1:**
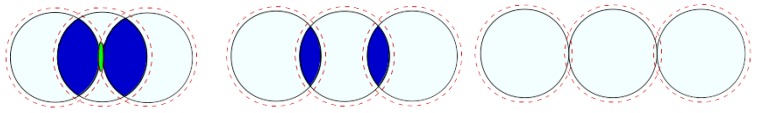
Stiff chains (**left**), semi-flexible chains (**middle**) and flexible chains (**right**) differ by the amount of overlap between adjacent spheres. The dashed red line indicates the range of the square-well attraction, the blue ellipses the inaccessible surface and the green ellipse the overlap of square wells of next-nearest neighbors along the chain.

In our model, semiflexible behavior is introduced by using steric hindrance. The polymer is modeled as a chain of tangent or fused hard spheres, as shown in Figure 1. The larger the overlap of the spheres, i.e., the smaller the ratio of bond length, *L*, to sphere diameter, *σ*, the more the bond angles are restricted to large values ($\vartheta_{\min}=2\sin^{-1}\left(1/2L\right))$. As a consequence, the chains get stiffer when the bond length is reduced, so we can employ the bond length as a parameter characterizing the stiffness of our chains. We choose σ=1 as our unit of length and vary the bond length in the range 0.51≤L≤1.

The case L=1 (shown on the right of Figure 1) is the limit of flexible chains, for which the phase diagram has been studied in detail before [29]. When we introduce an effective attraction between the monomers in the form of a square well potential,

(1) $$U\left(r_{ij}\right)=\left\{\begin{array}{cc} \infty & r_{ij}<1\\ -\varepsilon & 1<r_{ij}<\lambda \\ 0 & r_{ij}>\lambda \end{array}\right.,$$

the chain will collapse upon temperature reduction. We choose ε=1 as our unit of energy and temperatures are measured in units of $\varepsilon /k_{B}$. Although not the focus of the present work, the square-well potential can be used to study electrostatic interactions. For example, in [30], the phase behavior of charged colloids in solution is studied by mapping the screened Coulomb potential onto an effective square well attraction. Similarly, the square well chain model has been shown to provide a qualitatively valid representation for the thermodynamics of peptides in aqueous solution [31]. For λ<1.18 [32], the collapse is a first order transition in the thermodynamic limit, whereas for λ≥1.18, it is a second order transition followed by a first order crystallization transition at lower temperatures. With decreasing chain length, this crossover shifts to smaller *λ*, and for the chain lengths we study, the value of λ=1.1 that we employed in the simulation means that we have a second order collapse transition for the flexible case. With increase in the overlap (going from right to left in Figure 1), the chains get stiffer due to steric hindrance, and, at the same time, the accessible surface for non-bonded contacts gets smaller (semi-flexible regime with 0.55<L<1). For very short bonds, the square well attractions of next-nearest neighbors overlap leading to a permanent energy contribution (stiff regime with 0.51≤L≤0.55).

We have performed Stochastic Approximation Monte Carlo simulations (SAMC) [33,34], a version of flat-histogram Monte Carlo simulations [35], which we adapted to the simulation of polymer models [36,37,38]. These simulations determine a numerically exact (if the simulation is converged sufficiently) result for the density of states, g(E), of the model under study, or, more precisely, its logarithm, which is the micro-canonical entropy, S(E)=ln[g(E)], where we set $k_{B}=1$. In SAMC, as in Wang–Landau MC, the density of states is determined iteratively starting from an estimate $g_{0}\left(E\right)=1$ for the complete energy range, by performing an unbiased Monte Carlo simulation in configuration space and accepting new configurations with the acceptance probability

$$A\left(E\right)=\min\left(1,\frac{g(E_{\mathrm{old}})}{g(E_{\mathrm{new}})}\right).$$

The SAMC update of the current estimate for the logarithm of the density of states is [36]

(2) $$\ln\left[g\left(E\right)\right]=\ln\left[g\left(E\right)\right]+\gamma_{t}\left(\delta_{E,E^{\prime }}-1/M\right),$$

where *M* is the number of energy values or energy intervals for which one wants to estimate g(E). In each update, *M* is subtracted for all *E*, and only for the new energy $E^{\prime }$ (equal to either $E_{\mathrm{old}}$ or $E_{\mathrm{new}}$) accepted in the Monte Carlo procedure, the modification factor $\gamma_{t}$ is added to $\ln[g\left(E^{\prime }\right)]$. The following two necessary conditions [33,34] exist for this method to converge:

(3) $$\begin{array}{ccc} \sum_{t=1}^{\infty }\gamma_{t} & = & \infty , \end{array}$$

(4) $$\begin{array}{ccc} \sum_{t=1}^{\infty }\gamma_{t}^{\nu } & < & \infty \;\mathrm{for}\;\mathrm{some}\;\nu \in (1,2). \end{array}$$

From the density of state, one obtains, for instance, the canonical partition function as

(5) $$Z\left(T\right)=\sum_{x}\exp\left\{-\beta U\left(x\right)\right\}=\sum_{E}g\left(E\right)\exp\left\{-\beta E\right\},$$

where *x* is a point in configuration space, U(x) is the potential energy of the configuration and $\beta =1/k_{B}T$. Both g(E) and Z(T) contain the complete thermodynamic information on the model, and a determination of its thermodynamic properties is most efficiently performed in a combination of a micro-canonical (starting from g(E)) and canonical (starting from Z(T)) analysis [35]. We will analyze mainly the behavior of the derivative of the inverse micro-canonical temperature, $\gamma \left(E\right)=dT^{-1}\left(E\right)/dE=d^{2}S\left(E\right)/dE^{2}$ and of the canonical specific heat

(6) $$C_{N}\left(T\right)=\frac{2TZ^{\prime }Z+T^{2}ZZ^{\prime \prime }-T^{2}Z^{\prime 2}}{Z^{2}},$$

where the prime indicates a derivative with respect to *T*. The derivative of the micro-canonical temperature has peaks with a value larger than zero at first order transitions and peaks with a value smaller than zero at second order ones [39]. The canonical specific heat shows transitions as peaks and sometimes as shoulders, when transitions can not be well separated. A structural quantity that monitors the collapse transition is the radius of gyration of the chains. We obtain this—and all equilibrium observables—first as an arithmetic average, $\bar{R_{g}^{2}}\left(E\right)$, over visited configurations at a given energy in a simulation using the converged density of states, which can then be transformed into the canonical ensemble

(7) $$\langle R_{g}^{2}\rangle \left(T\right)=\frac{1}{Z(T)}\sum_{E}\bar{R_{g}^{2}}\left(E\right)g\left(E\right)\exp\left\{-\beta E\right\}.$$

## 3. Results

In the following, we will discuss pseudo phase diagrams for chains of length N=20 and N=40 as a function of bond length (i.e., stiffness) and temperature. As these are pseudo phase diagrams, they depend quantitatively (location of transitions) and qualitatively (order of the transition, number of transitions, resulting morphologies) on chain length. Such a study is therefore in spirit closer to the analysis of peptide pseudo phase behavior than to a study of the properties in the thermodynamic limit. The limiting behavior shows true phase transitions, but it loses pseudo phases of interesting morphologies. After all, chain stiffness is also a determining factor for the native state morphology of peptides and proteins.

### 3.1. Chain Length N=20

**Figure 2:**
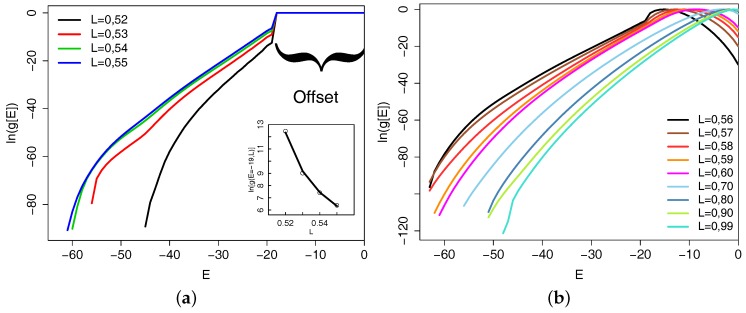
(**a**) shows the micro-canonical entropy for the stiff chains (note that, for N=20, we only studied bond lengths larger than 0.51); (**b**) for the semi-flexible chains. Note the shift in the energy spectrum in the stiff case due to the permanent overlap of interaction zones of next-nearest neighbors along the chain. The inset shows the jump at the step in ln[g(E)] and a fit with the law ${\left(L-0.5\right)}^{-3/4}$.

For this chain length, we start by presenting the basic result from our simulations, i.e., the micro-canonical entropy S(E)=ln[g(E)] in Figure 2.

For the stiff chains (0.52≤L≤0.55), there is a jump in the density of states at E=−18, which, for these chains, is the energy of permanently interacting next-nearest neighbors along the chain. The jump of the entropy seems to diverge for L=0.5, where the only possible bond angle is ϑ=π and the chain is a rigid rod (see inset on the left). For the semi-flexible chains (0.56≤L<1), a smeared out step is still there for L=0.56 but is smoothed out for larger *L*, while, at the same time, a maximum in the entropy occurs at finite energy. For energies larger than this value, the inverse micro-canonical temperature becomes negative, a well known behavior for the Boltzmann entropy of finite systems, which can be avoided by using the Gibbs definition of entropy [40,41].

**Figure 3:**
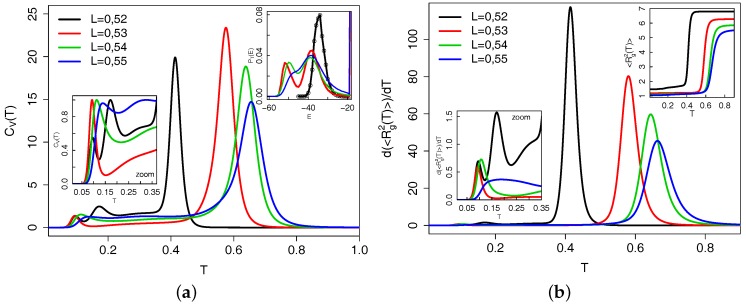
Part (**a**) shows the canonical specific heat for the stiff chains. The inset in the lower left corner shows the low temperature region in more detail. The inset in the upper right corner shows the probability distribution of the energy for the temperatures where the specific heat has a maximum. Part (**b**) shows the temperature derivative of the gyration radius for the same chains. The inset in the lower left corner highlights the low temperature behavior, the inset in the upper right corner shows the temperature dependence of the radius of gyration itself.

Although the entropy curves in Figure 2 contain the complete thermodynamics, information about pseudo phase transitions of the chain are buried in subtle curvature changes of these data. They become more clear when one calculates, e.g., the specific heat from them as is done for Figure 3a or determines the radius of gyration of the chains, for which its temperature derivative is shown in Figure 3b. Peaks in either of these quantities can be associated with structural transitions. The large peaks in the main panel in Figure 3b clearly locate the collapse transition of the chains, as one can also glean from the upper right inset of this figure, which shows the temperature dependence of the radius of gyration itself. As is well known for the collapse transition of polymers, the peak positions in different quantities signaling the transition are shifted with respect to each other. However, the main peaks agree well between the two quantities. The difference in peak positions, combined with information from other quantities, defines our error estimate for the determination of the transition temperature. The order of the transition can be obtained by looking at the energy histogram at the transition temperature, which is shown in the upper right inset in Figure 3a. For L=0.53 and L=0.54, we see a clear double peak structure indicating a first order pseudo phase transition. For L=0.55, the peaks are not well separated and the order of the transition is not well determined from this canonical analysis. A micro-canonical analysis of the peaks in the temperature derivative (as shown for N=40 in the next subsection) tells us that the L=0.55 collapse transition is of first order. The same is obtained for L=0.52, for which we have a highly asymmetric energy distribution due to the small available energy range for this bond length (see Figure 2).

**Figure 4:**
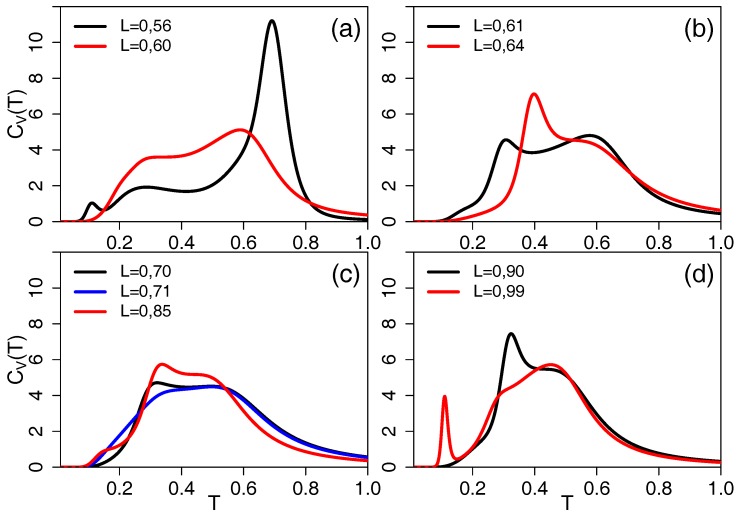
Canonical specific heat as a function of temperature for the bond length range of semi-flexible chains. (**a**–**d**) show different parts of this range.

The determination of the bond length dependence of the transition in the semi-flexible range is illustrated in Figure 4 using the canonical specific heat. For L=0.99 (panel d), a broad high temperature peak indicates the continuous collapse transition of this chain, while a shoulder and a sharp peak at lower temperature indicate further structural transitions. The low temperature crystallization of the flexible chain at L=1 is not well resolvable here because the chain is so short that the globule basically consists of the surface and can not undergo a further volume phase transition. For L=0.9, two peaks are visible with the lower temperature one being the main peak. Micro-canonical analysis indicates that this is a first order transition. In the range between L=0.7 and L=0.85, a broad plateau region develops between T≃0.3 and T≃0.6 (panel c), with the relative height between the high temperature and the low temperature part varying as a function of stiffness. For L<0.70, the peaks in the specific heat become again more pronounced (panel b) with the peak at higher temperature becoming the main transition for L≤0.61. For L=0.56 (shown in panel a), we cross over to the stiff chain behavior with a sharp and clear high temperature peak as in Figure 3a.

**Figure 5:**
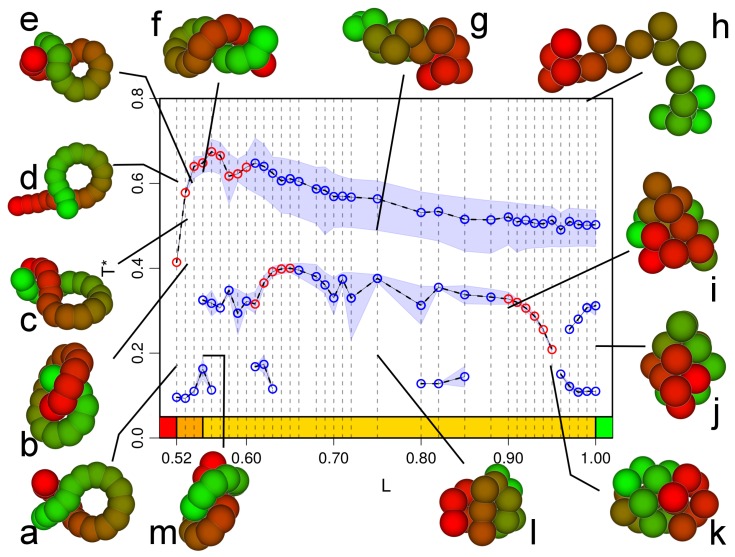
Pseudo phase diagram for chain length N=20. Blue symbols indicate second order transitions, red symbols indicate first order ones. The shaded areas are error estimates based on the different quantities analyzed. Isolated points are included when they occur in more than one observable and again error estimates are given. The configurations (**a**) to (**m**) indicate most probable configurations at a given bond length and temperature pair.

All the above analysis cumulates in the pseudo phase diagram of Figure 5. As expected for this chain length, the collapse transition is of second order for flexible and moderately semi-flexible chains, but it becomes a first order transition for L≃0.6. For all stiffnesses, we observe a regime of further structural transitions at lower temperature, which are of first order for two regimes of bond lengths. Further isolated points indicate local structural transitions which—for a short chain of length N=20—lead to observable features in the thermodynamic quantities, but which are not suspected to survive in the thermodynamic limit. For N→∞, the line of collapse transitions can be expected to become of first order for all stiffnesses, as it is already of first order for this choice of interaction range for the flexible chains of L=1, and stiffness tends to strengthen the first order character of the transition [15,16]. However, this conclusion is only valid if entropic stiffness (steric exclusion) like we introduce here has the same consequences as energetic stiffness (bending energy) studied in references [15,16]. The configuration snapshots included in Figure 5 show the transitions from random coil (h) to crystalline globule (j) on the flexible end. For semi-flexible chains (configurations g, i, k and l) the collapsed and ground states get elongated and some indications of lamellar packing (i and l) are observable. Stiff chain morphologies for L=0.52 above the “collapse” transition (d) and below it (a) are very similar and only differ by some winding of the chain ends around each other. For 0.53≤L≤0.55 (configurations b, c, e, f, and m), this winding gets easier as the chains are more flexible and tighter structures are formed. Which of the low temperature transitions and configurations survive for longer chains and what they signify is a different question. We will address this for a chain of length N=40, which still is in the “peptide” range in the next subsection.

### 3.2. Chain Length N=40

**Figure 6:**
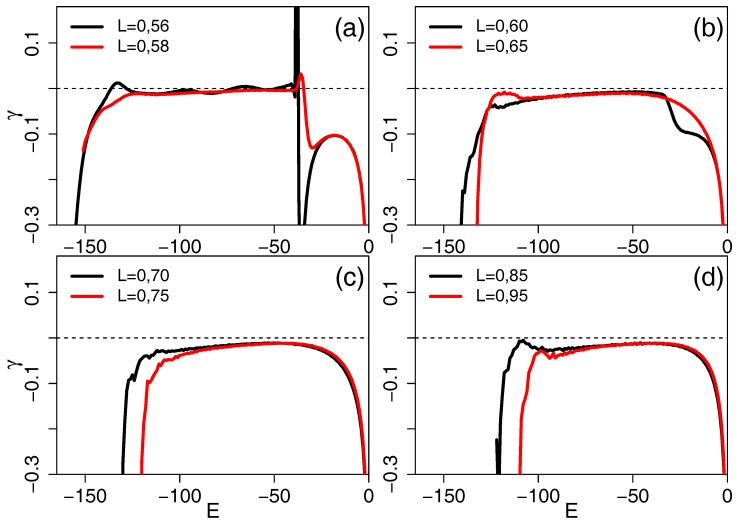
Second derivative of the entropy with respect to energy, $\gamma \left(E\right)=d^{2}S/dE^{2}$, for different bond lengths in the semi-flexible regime, going from the most flexible chains in (**d**) via increasing stiffness in (**c**) and (**b**) to the border of the stiff regime in (**a**). Peaks with heights above zero indicate a first order phase transition, the others indicate second order transitions.

For this chain length, we studied the same quantities as discussed in the last subsection for chain length N=20. Here, we illustrate the micro-canonical version of the analysis which is based on the second derivative of the entropy function, as shown in Figure 6 for chains in the semi-flexible range.

As a function of energy, we see (in panel d) a broad plateau region with a high energy (very broad) maximum for L=0.95 and L=0.85 with a value below zero, which indicates the second order collapse transition. At the low energy end of the plateau, both bond lengths exhibit a peak with a maximum below zero, indicating an additional second order transition. For L=0.75 and L=0.7 (panel c), this low energy feature splits in small oscillations of the curve, while, at L=0.65, it is visible again (panel b). For L=0.6 (panel b), we see a step at high energies, a remnant of the step in the entropy between E=−19 and E=−18, which gives rise to the high energy maximum seen in (panel a) for both L=0.58 and L=0.56. Both of these bond lengths also give a high energy peak which is larger than zero, indicating a first order transition. For L=0.56, another first order transition occurs at low energy, and two intermediate maxima very close to zero are barely visible in the intermediate energy regime.

**Figure 7:**
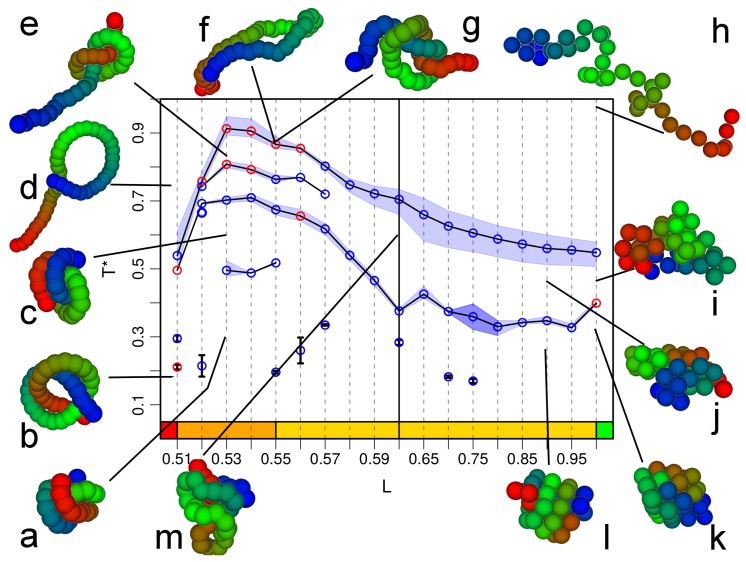
Pseudo phase diagram for chain length N=40 including examples for configurations (**a**–**m**) which are the most probable in the different regions. Blue symbols are for second order transitions, and red symbols are for first order transitions. The shaded regimes indicate error estimates coming from different observables. Only points resulting from a signal in more than one observable are included.

Combining the analyses of different micro-canonical and canonical observables, we obtain the pseudo phasediagram shown in Figure 7. Here, the regime of second order collapse transitions extends to smaller bond lengths (down to L=0.57) than for N=20. If the hypothesis that the entropic stiffness we are studying behaves similarly to the behavior found in the literature for energetic stiffness is valid, this would indicate that the approach to the thermodynamic limit of this pseudo phasediagram is not monotonous. However, as stated before, this hypothesis has not been proven yet. Below L=0.57, a further intermediate line of transitions occurs, which is first order for L≤0.54. For this chain, it is instructive to look at the equilibrium morphologies in the different parts of the phase diagram in more detail. Flexible chains at high energy (and temperature) are of course random coils (configuration *h*), which then collapse into liquid globules (configurations *i* and *j*), and, for L=1, collapse further into an fcc crystal (configuration *k*). For L=0.9, the low temperature configuration (*h*) shows no crystalline order but begins to show toroidal segments or helical structures also visible in configuration *m*, which is an intermediate temperature configuration at L=0.6. For L≤0.6, we observe the occurrence of knotted structures like those shown in configurations *a*, *b*, *c* and *g*, next to hairpin structures like configuration *f* and tennis racket like structures like configuration *d*, which occur in this bond length range at high temperatures at or above the collapse transition.

**Figure 8:**
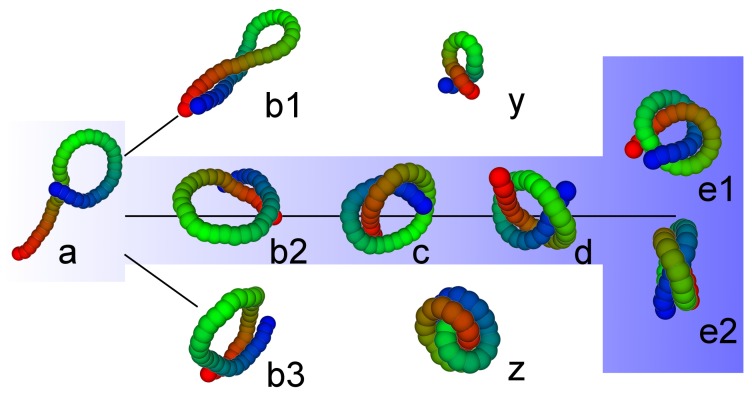
Path to the trefoil knot for N=40 and L=0.51 (**e1** and **e2** show two different views) starting from a tennis racket like configuration (**a**) when one reduces temperature (or energy) going from left to right through **b2**, **c** and **d**. Configurations **b1** and **b3** coexist with **b2**, configuration **y** is the ground state for N=20 and L=0.52, while **z** is the ground state for N=40 and L=0.52.

Configuration *b* is of special interest because it is a true trefoil knot. In this context, configuration *d* is also of interest because it is the first step on the path to the trefoil knot, which is shown in Figure 8. Starting from the tennis racket structure (*a* in Figure 8), the two ends slide over each other (b2) and start to twist around each other *c*. Finally, the knot tightens (*d*) and, for very low temperatures, the two ends come into contact (e1 and e2 show two views of a configuration slightly before that) and a closed trefoil knot is formed. Configurations b1 and b3 are degenerate in energy with b2. Configuration *y* shows the ground state for L=0.52 and N=20, which is close to configuration b1; the chain length N=20 is too short for the development of a knot at this stiffness, which is formed in the ground state for L=0.52 and N=40 shown in configuration *z*. The slightly larger flexibility for this bond length allows the knot to tighten and the ends to slip over each other.

**Figure 9:**
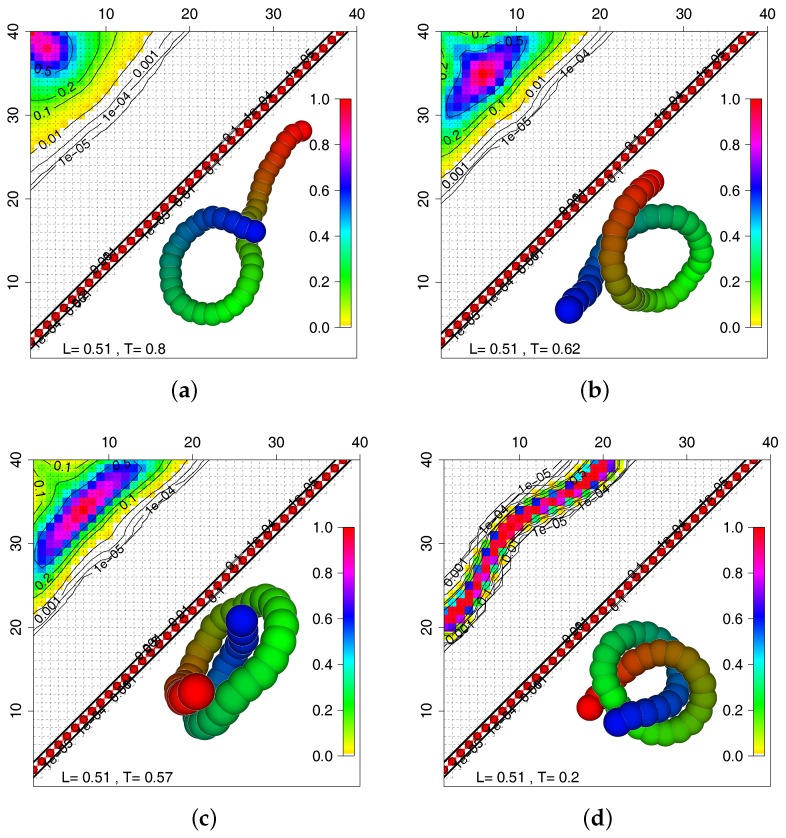
Contact matrices for the chain of length N=40, bond length L=0.51 and for the temperatures T=0.8 in (**a**); T=0.62 in (**b**); T=0.57 in (**c**) and T=0.2 in (**d**). The snapshots visualize the configurations giving rise to the contact matrices.

To characterize such a transition into a non-trivially ordered ground state like the trefoil knot more quantitatively, we make use of the contact matrix, a typical tool for the analysis of protein ground states. In the folded state, i.e., the ordered low temperature configuration, one has a fixed neighborhood for each monomer. The same is true for the trefoil knot. We have already shown that one can determine the temperature dependence of contact matrices from SAMC simulations in studies of a G$\bar{o}$ model [42] and get an idea about the formation process of the native state upon decreasing the temperature. In Figure 9, we show a sequence of contact matrices for the chain of length N=40 and temperatures going from T=0.8 in a, to T=0.62 in b, T=0.57 in c and T=0.2 in d. For all panels, due to the short bond length of L=0.51, there is always an off-diagonal next-nearest neighbor contact (n,n2), which is the diagonal of red dots. At the highest temperature, one has a high probability for an end contact forming (the snapshot shows one of the less probable contacts from the yellow regime). For smaller temperatures, the region of highest contact probability expands and moves inwards from the ends (panel b). The front boundary of the contact region, however, stays stationary as a function of temperature. For still lower temperatures (panel c), the region of high contact probability has extended into a strip parallel to the diagonal meaning that parallel chain segments at the two ends get into contact as shown in the snapshot. Finally, for the ground state, a well defined strip of neighbor contacts forms, indicating the unique native state (the trefoil knot) of this chain.

## 4. Discussion

In simulations of stiff chains with the persistent mechanism of flexibility [16,17,18,21,22,23], one determines phase behavior as a function of two energetic parameters, the temperature and the stiffness energy *κ*, taking the strength of the non-bonded attraction as the energy scale of the model. This can be done either at constant stiffness, i.e., constant κ/T as in [16,17,18] or at fixed stiffness energy *κ* like in [21,22,23]. The folded, toroidal and hairpin like structures (or tennis rackets) found in [21] were very similar to what was found at constant stiffness in [16,17,18], except for the explicit differentiation of multiply folded structures in [21]. Keeping the bond length constrained [22,23] but the rest of the model as in [21], one loses the toroidal structure but creates knotted configurations at intermediate stiffnesses for about the same chain length.

In contrast to these studies, stiffness in our model is introduced by steric effects, i.e., it is of entropic not energetic nature. Our phase diagram has only one energy parameter, while the second parameter (the bond length) is kept constant. Our very stiff chains can not fold back, and consequently the lamellar, folded structures are not found in the phase diagram. Hairpins and toroids exist at high stiffness, but they are no longer the ground state, which is a knotted structure, ideally the trefoil knot as for L=0.51 and N=40. The knotted structures in general have moved to the high stiffness region (they occur for L≤0.6) and are not found in the intermediate stiffness region, like in [22,23], where the high stiffness region is dominated by hairpins and folded chains. This comparison thus confirms our claim from the introduction: not all stiffnesses are created equal [7], and this is true even for models exhibiting the persistent mechanism of flexibility. There are common features in the phase diagram of stiff chains, but they are sufficiently different that one has to decide carefully which model really maps to a given stiff synthetic or bio-polymer. Finally, as we have also shown here, the pseudo phase diagram, of course, depends on chain length, such that structures which are dominant for one chain length might not be possible or be suppressed for another one.

## 5. Conclusions

We have presented flat histogram Monte Carlo simulations using the SAMC approach to determine the pseudo phase diagram of single semi-flexible chains as a function of stiffness and temperature. Our model consists of overlapping hard spheres interacting with a square well attraction. The degree of overlap determines the stiffness of the chains. In this aspect, the model is close to the behavior of real polymers, where typically the van der Waals radius of the atoms is almost twice as large as the bond length. We presented pseudo phase diagrams for chains of length N=20 and N=40, which exhibit different stable morphologies for low temperatures (or energies). The phase diagrams were determined from a combined canonical and micro-canonical analysis based on the micro-canonical entropy determined in the SAMC simulations. To extend the chain length range that can be studied, it might be useful to resort to the flat-histogram chain growth methods [43,44].

For our model, in which stiffness is induced by steric effects, i.e., it is of entropic origin, the ground state structures for stiff chains are knotted (similar to what was found by Marenz et al. [22] for intermediate stiffness in a model where stiffness is of energetic origin). We showed that, for the stiffest chain of length N=40, the ground state is given by an exact trefoil knot, where the two chain ends are in contact. We have established the route to the trefoil knot upon reducing temperature using contact matrices, a tool known from the analysis of protein configurations. Although the general types of collapsed and ground state morphologies observed in different models for semi-flexible polymers tend to be similar, the locations of different morphologies in the pseudo phase diagrams is model dependent. Thus, comparison with experiment must guide the appropriateness of using different mechanisms (e.g., energetic vs entropic) for chain stiffness. Noting that the pseudo phase diagrams are also chain length dependent, it is apparent that there exists a huge variety of possible collapse and folding behaviors to non-trivial morphologies resulting from the competition between chain stiffness and monomer attraction. Going beyond the homopolymer case [37,38] introduces further complexity on the path to protein-like polymers, for which the native state in addition is stabilized by specific interactions like, e.g., hydrogen bonds.
